# Understanding the Synthesis of Turbostratic/Flash Graphene via Joule Heating

**DOI:** 10.3390/ma18122892

**Published:** 2025-06-18

**Authors:** Faisal Mahmood, Christian Fabrice Magoua Mbeugang, Furqan Asghar, Xing Xie, Dan Lin, Dongjing Liu, Bin Li

**Affiliations:** 1 School of Energy and Power Engineering, Jiangsu University, Zhenjiang 212013, China; 2 School of Engineering, Anhui Agricultural University, Hefei 230036, China; 3 Department of Energy Systems Engineering, University of Agriculture Faisalabad, Faisalabad 38000, Pakistan

**Keywords:** Joule heating, turbostratic/flash graphene, structure, characterization, mechanism

## Abstract

The introduction of the Joule heating (JH) method for synthesizing turbostratic graphene has attracted considerable attention from researchers due to its promising potential for commercialization compared to earlier techniques. Numerous studies have outlined the technology’s basic operation and how parameters such as electric field, operating time, and temperature influence the quality and type of graphene produced. Despite this, there is still a lack of concise and comprehensive studies that exclusively focus on the JH method with turbostratic graphene as the target product. This review article is a facile attempt to provide the scientific community with an overview of the historical development and operational principles of Joule heating. It also discusses the structural and fundamental differences between turbostratic and conventional graphene, along with methodologies for characterizing turbostratic graphene. Furthermore, the synthesis mechanisms of turbostratic graphene via JH are analyzed, and the future perspectives for advancing this method are also presented.

## 1. A Brief History of Graphene and Its Structure

**Figure 1:**
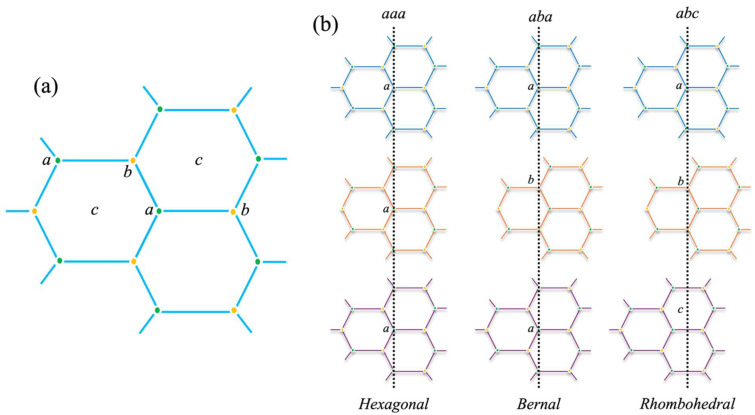
(**a**) Single lattice of graphene structure; *a* and *b* depict carbon atom sites. (**b**) Bi- and tri-layer graphene stacking types.

A single layer of graphene possesses a sp^2^ hybridized structure with a unit cell consisting of two carbon atoms and three in-plane sigma bonds (σ) per atom, which are exceptionally strong and are the backbone of the hexagonal structure. The two carbon atoms are located at the *a* and *b* positions, as displayed in Figure 1a [1]. This sigma bond exists in three out of the four valence electrons in a carbon atom present in graphene’s honeycomb-like lattice, which overlaps with the bordering carbon atom. This bond can be attributed to the resilience displayed by graphene’s lattice structure. A bi-layer graphene can essentially be categorized into three classes, *aaa* stacked, *aba* stacked, and twisted, depicted in Figure 1b [1,2], whereas tri-layer graphene can be stacked via three different arrangements. The sequences *aaa*, *aba*, and *abc* stacking denote the hexagonal, Bernal, and rhombohedral order, respectively [3], as represented in Figure 1b.

Studies have proven that graphene can be employed in multi-dimensional applications including energy storage, sensors, coatings, flexible electronics, catalysts, water purification, and displays, etc., as outlined in the existing literature [4,5]. Properties such as optical, electronic, and physical characteristics play a crucial role in the applicability of graphene. Mono-layered graphene can be employed where flexibility and average conductivity over large surfaces are required, i.e., in electronics, sensors, and solar cells. Presently, there is a tradeoff between the number of graphene film layers (i.e., transparency) and its electrical conductivity. A single layer of graphene has been reported to possess a resistance of almost 1000 Ω sq^−1^ and less resistance can be achieved with a higher number of layers [4]. Meanwhile, graphene in bulk powders can be synthesized in larger quantities compared to thin-area graphene films. Bulk graphene has proven to be useful in various domains such as material composites, energy storage materials, and lubricants. Here the morphology of the graphene sheet, its high surface area, and innate strength lead to significant enhancements. According to the requirement, scale, and design, graphene films and bulk graphene can be employed in multi-dimensional applications.

There are ample reviews available on the synthesis and applications of graphene through different techniques and substrates [4,5,6,7,8]. Therefore, this article will deviate from the conventional route of outlining the various processes (Table 1) that can be applied to transform various substrates into graphene. While emerging technologies such as atmospheric pressure plasma (APP) synthesis have also shown promise [9,10,11], they fall outside the primary focus of this review article. Instead, through this article, we attempt to delve into the Joule heating method and specifically focus on ‘turbostratic/flash graphene’. We aim to provide an overview of the Joule heating (JH) process, the characterization of turbostratic graphene, and the mechanism behind its synthesis.

## 2. Joule Effect and Heating: History and Applicability

In 1851, James Prescott Joule disseminated his findings in the form of a publication to the Royal Society of London [21]. His results suggested that heat released by the conductor, the current, the duration of current flow, and the resistance could be articulated by the formula *Q = I^2^Rt*, known as Joule’s Law. Q denotes heat in Joules (J), I denote the current in amperes (A), the resistance is considered in ohms (O) expressed by R, and t represents the time of the passage of current recorded in seconds (s). Through the Joule effect, it can be observed that electrical energy can be converted to heat energy; this is one of the main fundamental concepts of thermodynamics and has immense importance and applicability in various domains. In normal electrical systems, the Joule effect is considered an adverse byproduct [22]. This effect is clearly visible in conductors like supercapacitors, batteries, and wires where the materials when faced with an electrical current generate heat (i.e., thermal energy) due to their internal resistance [23]. This not only damages the electrical components but also poses a safety risk. Due to the rapid heat generation, with minimum pollution and high efficiency, the Joule effect is employed in multi-dimensional domains such as heating in rice cookers, electric irons, and electrical furnaces. It is also utilized during metal smelting, in resistance welding, and even in medical therapy through therapeutic devices. In material science, Joule heating allows to cater to a specific environment where reactions requiring very high temperatures in a short time can be carried out to synthesize certain materials. The application of Joule heating in material sciences has been studied during the last few decades and has made substantial progress while demonstrating its potential [24]. Joule heating is a rapid technique through which controlled uniform and high temperatures can be achieved, which leads to the enhancement in the materials’ structure and performance and, hence, a novel route to synthesize materials in domains of ceramics [25,26], metal powder metallurgy [27], fabricating nanoparticles [28], and flash graphene [20].

Joule heating does not require furnaces and is not affected by reaction solvents and gases; this alleviates energy consumption and pollution challenges as compared to conventional pathways to synthesize graphene [20,29]. This process has very rapid heating and cooling rates (tens to hundreds of degrees Celsius per second) with a very high targeted final temperature. The reaction residence time is also very short (less than 10 s); this leads to the overall process being less energy intensive and resulting in higher energy efficiency than conventional heat-oriented techniques [30,31]. The very short processing time due to the rapid heating and cooling mitigates redundant thermal consequences such as fluctuations in structure due to temperature gradients, particle sintering, and material oxidation [32,33]. Furthermore, due to the rapid heating and cooling of the JH method, the process exhibits a higher heat transfer with less heat loss and does not require a network to transfer the heat when compared to conventional heating methods.

## 3. How Does It Work and How It Is Applied

### 3.1. Plate-like Electrode Configuration

**Figure 2:**
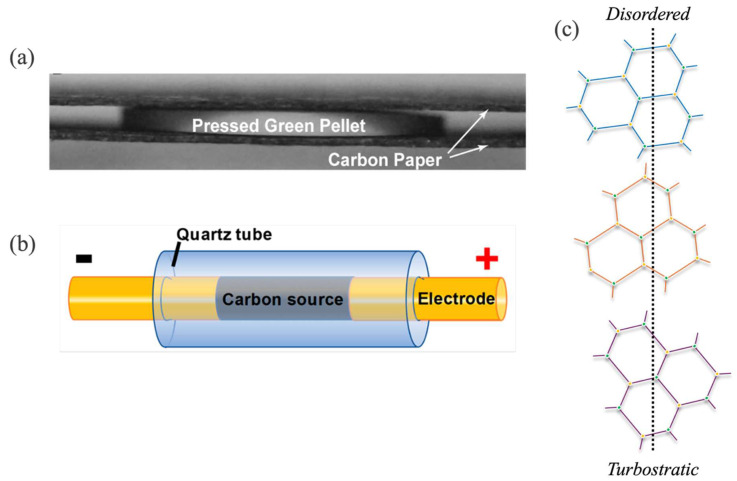
(**a**) Schematic diagram of the JH process by sandwiching the feedstock between two carbon fabric/strips. (**b**) Applying JH by employing the electrodes as rods. (**c**) Structure of turbostratic graphene showcasing misaligned layers synthesized by the Joule heating method. (**a**) Reproduced with permission from ref. [25]. Copyright 2020 Science. (**b**) Reproduced with permission from ref. [34]. Copyright 2020 American Chemical Society.

The Joule heating (JH) method can be carried out in two distinct ways in accordance with the heating method. One way to perform Joule heating is to sandwich the substrate in between two thin fabrics or strips made of carbon black (Figure 2a). In this setup, when the electric current is discharged, the material’s internal resistance results in the conversion of the electric energy to heat. This heat rapidly increases the temperature of the carbon strips/fabric and through thermal radiation, this heat is transferred to the material being processed, hence the rapid thermal treatment of the substrate. This instantaneous effect of heating can induce ultrafine structure and can be employed for value-added purposes of carbon substrates.

In a study conducted at 900 °C by Zhang et al. [35], they were able to apply this phenomenon to activated carbon and achieve a high ultrafine structure along with the presence of abundant surface functional groups. JH not only facilitates the formation of an ultrafine structure but also retains a majority of the functional groups from the substrate as it does not require any post-processing treatment such as acid washing. Furthermore, JH can also be employed with conventional activation chemicals to achieve a more refined porous structure. This was reported by a study performed by Liu et al. [29], where they processed a blend of carbon substrate with KOH and exposed it to JH reaching a maximum temperature of 900 °C; the ultrafast heating and cooling led to the formation of a more refined and porous structure. Activation in a conventional furnace leads to the slow formation of small drops of melted KOH, due to the prolonged reaction time. This reduction in the Gibbs free energy of the system causes the condensation of the small drops into large drops of KOH, leading to the generation of a large and non-uniform distribution of pores. Furthermore, the employment of carbon strips/paper has also been applied for sintering ceramics [25].

### 3.2. Rod-like Electrode Configuration

Another route to utilize JH is to employ the carbon substrate as the conductor. If the feedstock is non-conducive in nature, a portion of conductive substance such as carbon black is incorporated to facilitate the flow of current through the substrate, as depicted in Figure 2b. This is done to maintain the resistance of the substrate between 1 and 1000 Ω. By discharging a large capacitor bank in milliseconds, the substrates can be thermally treated instantly at extreme temperatures. The discharge time is controlled by utilizing relays having a delay time of up to milliseconds. The quality and type of graphene obtained from JH can be affected by operating parameters such as electric field and electric shock time. Eddy et al. [36] reported that the JH process can be employed to complete a three-phase transition process. In this step, a carbonaceous substrate or amorphous carbon is transformed into turbostratic graphene having a disordered structure (Figure 2c). By adjusting the current or its pulse time, turbostratic graphene can further be transformed into ordered graphene or graphite exhibiting *aba* or *abc* stacked layers, as shown in Figure 1b. It was also concluded that carbon substrates’ own electric field, which is amplified by the passing current, facilitates the transformation process by reducing the activation energy required for the process. Furthermore, the electric current energetically facilitates the orientation of the layers in the direction of the applied electric field and influences the graphitic lattice in a way that reduces the interlayer spacing [36]. During JH, most of the generated heat is dissipated in the form of black body radiation [20], proven by very bright flashes of light, leading to the synthesis of graphene. The reaction temperature by adopting this route of JH can reach around 3000 °C within a very short span. JH can be considered an improved version of direct laser writing (DLW) to synthesize graphene. DLW is a versatile technique where different types of feedstocks can be easily processed from laser-induced graphene (LIG) [14,37,38,39]. DLW results in a rapid increase in temperature and pressure but is limited due to the area where the laser is bombarded. The instant rise in localized temperature and pressure facilitates the volatiles to escape and the carbon is left to rapidly quench, leading to the formation of graphene. Keeping this in mind, FJH was applied by Loung et al. [20], where graphene was produced in bulk amounts and was characterized as turbostratic or flash graphene, which exhibits a disoriented stacking of layers in graphene. The group reported a yield of 80 to 90% with a purity of almost 99% requiring no post-process treatment and, hence, the enhancement of production efficiency.

**Figure 3:**
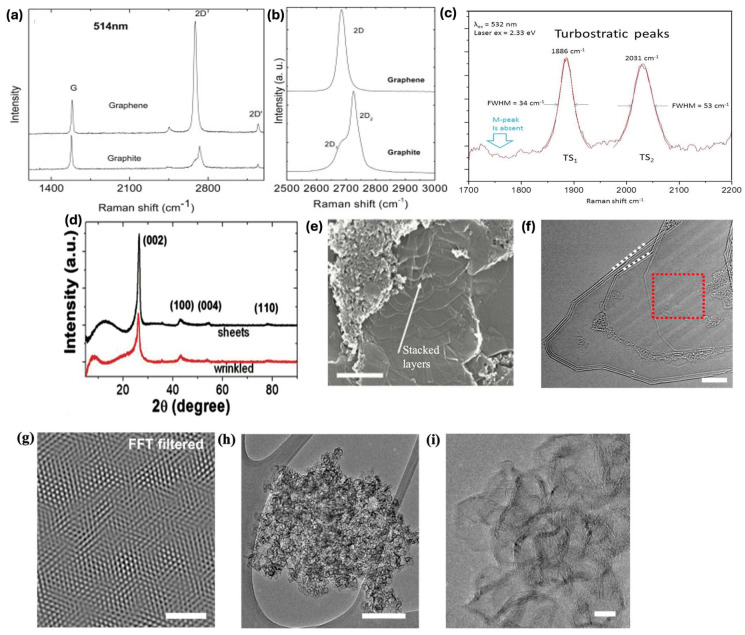
(**a**) Raman spectra comparison obtained at 514.5 nm for graphene and graphite. (**b**) 2D peak compared for graphene and graphite. (**c**) Raman spectra at high resolution confirming stacking with turbostratic graphene. (**d**) Morphology of powder flash graphene from XRD analysis. (**e**) SEM image of turbostratic graphene sheets, which can be exfoliated (the scale is 500 nm). (**f**) TEM image of turbostratic graphene displaying striations, which represent rotational mismatch; scale is 5 nm. (**g**) Inset region FFt filtered image of the red highlighted region showcasing a moiré pattern; the scale is 2 nm. (**h**) Wrinkled graphene prepared by JH method observed by a TEM image; the scale is 200 nm. (**i**) HRTEM image of the synthesized wrinkled graphene; scale is 10 nm. (**a**,**b**) Reproduced with permission from ref. [41]. Copyright 2007 Elsevier. (**c**) Reproduced with permission from ref. [20]. Copyright 2020 Springer Nature. (**e**–**i**) Reproduced with permission from ref. [34]. Copyright 2020 American Chemical Society.

When the cleavage of bonds occurs at very high temperatures and is reordered in a very short time, they thermodynamically form sp^2^-hybridization into graphene sheets. Graphene is the most stable form of carbon and due to the rapid effect of the electric current, the formed graphene sheets do not have ample time to properly stack into *aba* ordered or stable layers of graphite, as shown in Figure 1b. Instead, the resulting graphene layers are arranged in a metastable state termed turbostratic or flash graphene due to the ‘flash’ nature of the process or the applied electric current. It can be observed that areas with low electron density, i.e., the middle area of the benzoid ring, overlap with areas of high electron density, which are the edges of the ring (Figure 1b). Due to this arrangement, there is significant electrostatic interaction between the stacked layers of graphene, leading to the exfoliation of these layers being a tedious venture. For turbostratic graphene, the layers are disordered, so it is relatively easy to separate the misaligned layers [40] (Figure 2c). When the voltage is applied, the current will flow through the path of least resistance within the substrate material. This leads to the supply of large amounts of heat to the materials conducting particles. The particles lying within the path of the current flow and its surrounding area would be graphitized and annealed, increasing conductance. The localized defects exhibiting less conductance would continue to be heated and annealed, leading to the elimination of defects. Due to the rapid heat treatment and abrupt annealing, the complete material would be transformed, resulting in highly conductive layers of turbostratic graphene facilitated by the free amorphous carbon atoms. The substrate particles, which do not lye directly in the conductance path, are assumed to undergo limited graphitization and arrange themselves in the form of wrinkled graphene, as characterized in Figure 3d. Due to this, flash graphene has low interlayer interaction, leading to higher dispersibility in water and organic solvents, which was demonstrated by Loung et al. [20].

## 4. Characterizing Turbostratic/Flash Graphene

### 4.1. Raman Spectroscopy

Raman spectroscopy is widely utilized and is considered the approved and standardized technique to characterize graphene, as a single spectrum from it can provide vast information. Using Raman spectroscopy to characterize and analyze graphene has been performed in ample studies [40,42,43]. Information such as quality and symmetry, along the stacking arrangement of the neighboring graphene sheets, can be extracted using this technique [44]. The optical and electrical properties of graphene are attributed to the rotational disorder within the graphene sheets. This misalignment of graphene sheets can be confirmed using Raman spectroscopy. A Raman spectra turbostratic graphene normally exhibits three peaks termed D, G, and 2D (also labeled as *G’*). As Raman spectroscopy is a localized analysis technique, the intensity of these peaks may vary greatly at different regions within the same sample. By determining the full of at half maximum (FWHM) or the ratio between 2D/G peak intensities, we can assess graphene’s layering, but this cannot be considered for turbostratic systems. As previously mentioned, the layers in turbostratic graphene adhere to each other, so the collected spectra remain independent or unaffected by the number of layers where the 2D peak is observed to be Lorentzian in shape and narrow, as detailed in a study carried out by Ferrari A. [41], shown in Figure 3a. As turbostratic graphene possesses weak interlayer interactions, its 2D peak benefits from the double resonance enhancement and its intensity is greatly enhanced. Meanwhile, in the case of a reverse *ab* stacked graphite, its 2D peak does not possess a high intensity and loses its Lorentzian shape with regard to the number of graphene sheets, as displayed in Figure 3b. In turbostratic graphene, the intensity of the 2D peak may increase if there are more layers. The 2D/G ratio has been observed to be as high as 17 for turbostratic graphene [20]; however, ratios of higher than 2 are normally observed [41]. This observed value is opposite to the usual thought that the highest value obtainable for the 2D/G ratio is 4, which is observed from a single layer of graphene grown by the chemical vapor deposition (CVD) method.

From the Raman spectroscopy, other less-known and weak peaks may also be observed which are termed TS_1_, TS_2_, and M peaks. These peaks are crucial in understanding the rotational alignment in turbostratic graphene [45,46,47]. When an excitation source of 532 nm is employed, the TS_1_ (1880 cm^−1^) and TS_2_ (2030 cm^−1^) peaks appear to be approximately 30 times less intense as compared to the G peak. By applying peak fitting, the TS_1_ peak was fitted with one Lorentzian having an FWHM of 35 cm^−1,^ and the TS_2_ possessed two Lorentzians showcasing an FWHM of 52 cm^−1^. Using the same excitation source, the M peak occurs at 1750 cm^−1^, which is 25 times less intense in comparison to the G peak. The cited studies [45,46,47] state the M peak is a result of combining modes of multiple phonons, which appears due to the coupling between sheets and is an indication of the *aba* stacking of multiple graphene layers, as depicted in Figure 1b. However, the turbostratic graphene analyzed by Luong et al. [20] displayed disrupted or misaligned graphene sheets; therefore, the M peak was not observed as displayed in Figure 3c. Hence, turbostratic graphene should possess TS_1_ and TS_2_ peaks, but the absence of the M peak confirms the misstacking of the graphene sheets, indicating disordered graphene layers. Therefore, Raman spectroscopy can be a very useful tool for not only characterizing regular graphene but also for turbostratic graphene possessing misaligned or misoriented graphene sheets. However, no matter the method, whether it is top-down or bottom-up, the graphene synthesized will be dispersed and have a range of quality that varies from region to region. Displaying and analyzing a single spectra of Raman from a specific region of the sample can be misleading. This can be avoided by providing an average spectrum obtained by performing Raman spectroscopy at different regions of the sample. Another way to disseminate proper Raman information is through scatter plots exhibiting the intensity ratios of the main peaks, thereby aiding in analyzing a larger area of the synthesized graphene sample. By doing this, Raman spectroscopy can be employed to enhance and improve the reproducibility of various graphene synthesis methods.

### 4.2. X-Ray Diffraction (XRD)

X-ray diffraction is a non-destructive characterization technique widely employed to analyze the structure and orientation of graphene. This is done by observing the diffraction of the incident X-rays on the surface of the sample [48]. XRD is a useful technique as it can determine graphene’s crystalline structure and its orientation, and it is also able to precisely observe its lattice constants. It can also be used to calculate the interlayer spacing (d) by measuring change in 2θ (2 theta) by using Bragg’s equation. The complete transformation of the carbon feedstock/substrate to crystalline graphene can be observed through the XRD pattern. An intense peak at 26.3° (002) can be seen, denoting an interlayer spacing of almost 3.45 A°, which is considerably larger than the 3.36 A° for graphite. For turbostratic graphene, the (002) peak has a larger FWHM; its symmetry leans towards lower diffraction angles, and it also exhibits a weak (100) peak (Figure 3d), which are its main characteristics [49,50]. As the (002) peak is broader in wrinkled graphene, this is an indication of a larger crystallite size along the c-axis. This indicates a wider arrangement of stacked layers as compared to regular graphene, as displayed by the scanning electron microscope (SEM) depicted by the white arrow in Figure 3e. Apart from the most commonly used Raman spectroscopy and XRD techniques, transmission electron microscope (TEM) and SEM are also employed to complement and confirm graphene’s presence and its morphology [20,34,38].

### 4.3. Transmission Emission Microscopy (TEM)

The TEM image in Figure 3f displays different sheets of turbostratic graphene exhibiting striations suggesting rotational incongruity. The disordered rotation between the graphene sheets (indicated by white dotted lines) exhibits a moiré pattern. This pattern is observed as striations only when the graphene layers are misaligned. The red highlighted portion was further analyzed and is displayed in Figure 3g, where the moiré pattern is more prominent due to the misalignment of the stacked layers, as depicted in Figure 2c. In a study performed by Stanford et al. [48], it was reported that graphene synthesized through the JH method consists of turbostratic graphene and small graphitic carbon particles. An HRTEM image of the small carbon particles is depicted in Figure 3h,i. These particles were graphitic in nature, possessing many bends, and have been reported to exhibit a thickness of 3 to 8 layers [20]. These non-graphitized carbon particles [34,50] were labeled as wrinkled graphene, showcasing an interatomic spacing of almost 0.34 nm; this spacing relates to the value obtained for turbostratic graphene with the absence of *aba* stacking (Figure 1b). A comparison has been summarized between graphene synthesized from conventional methods stated in Table 2 and turbostratic/flash graphene prepared through the mentioned JH method.

## 5. How Turbostratic Graphene Is Synthesized (Mechanism)

### 5.1. Simulation and Experimental Insights

To understand the synthesis of flash or turbostratic graphene through the JH method, researchers have employed simulation techniques such as molecular dynamics (MD) or density functional theory (DFT). They have been used to analyze the structure of carbon-based materials and how they transform during the treatment at elevated temperatures. For the JH process, simulations have been performed to investigate the graphitization and annealing of the precursors within milliseconds. The simulations have revealed that the operating temperature and the density of the carbon atoms have a crucial effect on the annealing process and the final ratio of sp^2^/sp^3^ after the annealing process.

**Figure 4:**
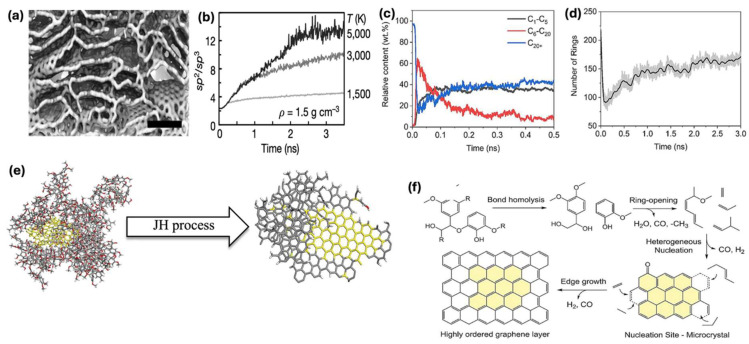
(**a**) Molecular dynamic simulations results displaying ordered graphene layers after annealing at 3000 K; scale is 1.5 nm. (**b**) Variation in sp^2^/sp^3^ hybridization ratios with respect to JH process time for a material having a density of 1.5 g cm^−1^. (**c**) Amount of content of various chemical species with the passage of JH processing time. (**d**) Trend of the number of aromatic rings at varying JH process times. (**e**) Simulation of JH processing 10 lignin molecules and 1 carbon black molecule (yellow) and their realignment post-bond cleavage. (**f**) Proposed mechanism of graphene from synthesis by processing lignin through JH method. (**a**,**b**) Reproduced with permission from ref. [20]. Copyright 2020 Springer Nature. (**c**–**f**) Reproduced with permission from ref. [53]. Copyright 2025 Elsevier.

In a study performed by Luong et al. [20], simulations were performed on amorphous carbon with varying operating temperatures. The obtained computational data agreed well with the performed experiments, which proved the correlation between the operating temperature with the formation of graphene and defect healing. The structure obtained from simulations is displayed in Figure 4a along with the change in hybridization presented in Figure 4b. The experiments proved that the synthesis of high-quality graphene was possible by rapid thermal treatment with temperatures higher than 3000 K with a time span of almost 5 ms. The results from the performed simulations were not able to verify the formation of turbostratic or graphene displaying disordered layers. The simulations were limited to less than 5 ns due to computational constraints. Due to these limitations, the simulation study was not able to confirm the synthesis of turbostratic graphene with disordered layers (Figure 2c), which occurs due to the rapid quenching step, making it impossible for layers to stack in the *aba* order, as presented in Figure 1b. Meanwhile, it has been reported that by increasing the JH time to 5 s, the formation of graphene layers is more aligned with the *aba* order [20]. The study stated that a single crystal of turbostratic graphene in nature, when faced with thermal treatment, facilitates the growth of graphene. These crystals or ‘seeds’ then merge and facilitate the growth of graphene layers. The Raman analysis reveals that a longer JH time leads to the stacking of layers in the *aba* order. This further validates the assumption that instant heating and cooling are crucial to kinetically trapping the turbostratic layers of graphene. The simulation was also performed at 3500 K, which again revealed the increase in sp^2^ hybridization (Figure 4b), but the results failed to display discrete sheets of turbostratic graphene. The annealing time set in the simulation study was only about 0.002% of the actual time the atoms of the amorphous carbon experience during the JH annealing process. Along with this, the lack of other factors such as resistance, fluctuations in charge, or electric current during the simulation may contribute to the presented JH mechanism. The presented experimental and simulation study provides a basic understanding of the synthesis of turbostratic graphene through the JH method. As it was performed on amorphous carbon, it can be said that it fails to account for the presence of impurities or various volatiles that may arise during the processing of carbon precursors such as biomass. Therefore, impurities or unwanted volatiles may also influence the formation of graphene.

### 5.2. Structural Transformation of Amorphous Carbon to Turbostratic Graphene

The transformation of amorphous carbon to turbostratic graphene during the Joule heating method is primarily governed by a rapid increase in temperature and short processing time. Thermal energy from the high operating temperature, i.e., 3000 K, facilitates rearranging the carbon atoms from disordered to an ordered configuration. However, due to the rapid processing times and rapid quenching involved, the carbon layers do not have sufficient time to align in a thermodynamically stable state (i.e., aba staking), as displayed in Figure 1. Instead, the carbon layers are misoriented, resulting in the formation of turbostratic graphene. This turbostratic structure of graphene is characterized by random rotational stacked layers and possesses a larger interlayer spacing (~3.44 Å) as compared to graphite, which has an interlayer spacing of ~3.35 Å. This larger interlayer spacing results in weaker van der Waals interactions and alters electronic and mechanical properties. Loung et al. [20] confirmed that by modifying the operating parameters such as heating rate, process duration, and heating rate, it is possible to tune the extent of the disorder and the interlayer spacing. A high heating rate and rapid quenching are crucial to kinetically trapping disordered stacked layers and can suppress the formation of graphene-like order. This ability to regulate the stacking of carbon layers through thermal dynamics is core to synthesizing turbostratic structure instead of conventional ordered structure.

In a study performed by Guan et al. [34], lignin was taken as the carbon precursor and transformed to flash graphene through the JH method. Along with the experimental study, they performed MD simulations on the process. The simulations were carried out on a system consisting of 10 lignin molecules and 1 carbon black (CB) particle (Figure 4e). They report that around the 5 ns mark, the amount of content of various species begins to stabilize (Figure 4c), where species within the C_6_-C_20_ range account for 10% while C_1_-C_5_ and C_20+_ reach up to 40%. It was observed that initially, the total number of rings decreased and then increased (Figure 4d). This can be attributed to the cleavage of aromatic rings at a severe process temperature of 3000 K. The cleavage was followed by realignment and polycondensation, resulting in aromatic clusters and, hence, leading to the increase in the total number of rings. The study revealed that the synthesis of lignin-derived graphene was facilitated by the presence of carbon black particles/crystals, which provided the basis for the arrangement of ordered layers, as depicted in Figure 4f. The breakage of bonds within the lignin structure provided free carbon atoms, which were attracted to the CB crystals and began to align forming graphene layers.

To recapitulate, if the substrate is conductive, during the process, the free carbon atoms are attracted to graphitized carbon particles serving as ‘seed’ or ‘nucleation sites’ for the growth of graphene layers. If the substrate is non-conductive and carbon black is added, then its particles serve not only to enhance the conductance but also as nucleation sites for the growth of graphene layers [54,55]. The rapid quenching step in both scenarios influences if the growing graphene layers will form ordered or misaligned layers resulting in conventional stacked graphene (Figure 1b) or turbostratic graphene (Figure 2c), respectively.

## 6. Perspective and Future Recommendations

The JH method has proven to be an invaluable technique in synthesizing graphene; it provides a promising research direction in its employment in situ preparation of graphene-based composites. This could be perceived by introducing precursors such as metal–organic frameworks (MOFs) or metal oxides into the carbon feedstocks. The rapid and severe thermal environment provided by JH could facilitate concurrently reducing and decomposing the precursors and lead to the formation of synergistic stable structures. These materials may exhibit enhanced performance in domains such as energy storage, heterogeneous catalysts, and electrochemical sensing resulting from their unique tailored morphology, functional surface chemistry, and high conductivity. Although JH has been commercialized by Universal Materials Inc. in Canada [4], there still exists room for the technology to further mature. It can be modified or enhanced by making the process continuous or semi-continuous. The conceptual design could lead to a rotary system or conductive belt-driven platforms enabling the uninterrupted processing and feeding of raw materials. Such systems would have optimized control over thermal parameters, improved energy efficiency, and overall significant production output. Further investigation towards the development and configuring of such scalable reactors is crucial to overcome constraints attached to batch processing and for the utilization of the JH’s full potential.

Another avenue with ample opportunities lies in the targeted alteration of structure in terms of porosity and doping of graphene during the JH process. Biomass-derived feedstocks enriched with desired heteroatoms such as phosphorous, sulfur, or nitrogen—naturally occurring amino acids, proteins, or lignin—can serve as sources for the dopant elements. The unique environment during the JH process can promote the incorporation of the atoms into the graphene lattice, yielding stable materials exhibiting modulated electronic structures. Such materials will be highly desirable in applications where tunable electronic properties and surface functionalities are required. The synthesis of these materials can further be optimized by the data-driven approach employed for the selection of feedstocks, leading to further enhancement of the utilization of the JH method. The development of predictive models or machine learning algorithms that can correlate specific substrate characteristics (e.g., volatile content, impurities, and carbon-to-oxygen ratio) with quality, yield, and structural features of the resulting graphene-based materials could provide new avenues to investigate material design. The development of such models would reduce the dependence on conventional optimization and accelerate the discovery of viable feedstocks varying from biomass to waste resources.

To ensure the purity and consistency of the synthesized graphene products and their applications with strict quality requirements, the integration of post-synthesis purification strategies into the JH workflow is recommended. Techniques such as electrostatic separation, inert gas purging, or solvent extraction could be developed, modified, and integrated for the removal of residual ash, metal impurities, or other non-desirable by-products. The implementation of such techniques in real time would allow to preserve the throughput advantages of the JH process while enhancing the suitability of the synthesized products aimed toward high-end applications such as biomedicine and electronics. Furthermore, the transformation of JH-based graphene into printable formats such as conductive inks or pastes presents a strategic pathway for commercialization. Although turbostratic graphene from the JH process is dispersible, further optimization of the dispersion ability, rheological behavior, and adhesion properties—preferably by employing environmental benign solvents—could enable further the penetration of graphene into flexible electronics, wearable sensors, and printed circuits. This would circumvent complex and tedious post-treatment and align with the trends and demand of low-cost electronics and additive manufacturing.

Lastly, the avenue of feedstock blending warrants further investigation. The simultaneous processing of diversified carbon sources, such as lignocellulosic biomass and synthetic polymeric wastes, may offer a pathway for tunable structural, electronic, and surface properties of the end graphene-based products. However, the compositional heterogeneity of such mixtures presents challenges in terms of process control and purification. Systemic studies could elucidate the thermochemical interactions under the severe and abrupt JH conditions, which will enable rational design of multi-functional graphene materials tailored for specific applications in multi-dimensional domains.

## Figures and Tables

**Table 1:** Comparison of various processes for the synthesis of graphene.

Method	Product	Layer Control	Scalability	Cost	Quality	Ref.
Chemical Vapor Deposition (CVD)	Thin films	Excellent (mono–few-layer)	Moderate	High	High (electronic grade)	[12]
Epitaxial Growth on SiC	Thin films	Good (few-layer)	Low	Very high	High	[13]
Laser-Induced Graphene (LIG)	Thin films/patterns	Poor–Moderate (multi-layer)	Moderate–High	Low–Moderate	Moderate–High	[14]
Spray/Spin Coating (from Graphene oxide (GO)/reduced GO (rGO)	Thin films	Poor (multi-layer)	High	Low	Low–Moderate	[15]
Liquid Phase Exfoliation (LPE)	Bulk	Poor (multi-layer)	High	Low	Moderate	[16]
Electrochemical Exfoliation	Bulk	Poor (few–multi-layer)	High	Low	Moderate	[17]
Chemical Reduction of GO	Bulk	Poor (multi-layer)	High	Low	Low–Moderate	[18]
Atmospheric Pressure Plasma (APP)	Thin films/bulk	Good (few–multi-layer)	High	Moderate	Moderate–High	[19]
Joule Heating (Flash Graphene)	Bulk	Poor (multi-layer)	Moderate	Low–Moderate	Moderate–High	[20]

**Table 2:** A comparison of various properties between turbostratic graphene and graphene synthesized from other methods.

Property	Flash/ Turbostratic Graphene [20]	Chemically Derived Graphene [18]	Laser-Induced Graphene (LIG) [14]	CVD Graphene [51]	Mechanically Exfoliated Graphene [52]
Production Method	Flash Joule heating; rapid pyrolysis of carbon feedstocks	Chemical reduction of graphene oxide (GO/rGO)	Laser writing of carbon precursors	Thermal decomposition of hydrocarbons on metal substrates	Mechanical peeling of graphite
Layer Stacking	Disordered; rotationally misaligned (turbostratic)	Typically, multi-layer with some disorder	Multi-layer; often porous and disordered	AB-stacked (Bernal)	Single to few-layer, typically high-order
Interlayer Coupling	Weak (due to misalignment)	Moderate to weak, varies with reduction quality	Weak–moderate due to porosity	Strong π–π interactions	Strong
Electrical Conductivity	High in-plane due to decoupling	Moderate (~10^2^–10^3^ S cm^−1^)	Moderate (~10^2^–10^3^ S cm^−1^)	Very high (~10^4^ S cm^−1^)	Very high
Thermal Conductivity	High (variable with defects; up to ~2000 W m^−1^·K^−1^)	Lower (~100–500 W m^−1^·K^−1^)	Moderate–high (material-dependent)	Very high (~5000 W m^−1^·K^−1^ for monolayer)	Very high
Ease of Exfoliation	Easy due to weak interlayer bonding	Not applicable (not exfoliated)	Not applicable (direct-write process)	Hard (monolayer already formed on substrate)	Moderate (labor-intensive)
Crystallinity	Moderate; more defects than pristine graphene	Moderate; defect sites remain after reduction	Moderate; influenced by laser parameters	High; nearly defect-free in optimized systems	High
Cost and Scalability	Low cost; highly scalable from waste/biomass	Low cost; highly scalable	Low–moderate cost; highly scalable	High cost; moderate scalability due to CVD equipment	High cost; limited scalability
Applications	Batteries, supercapacitors, EMI shielding, composites	Flexible electronics, inks, coatings	Sensors, energy storage, antimicrobial coatings	High-performance electronics, sensors, flexible displays	Quantum devices, metrology standards

## Data Availability

No new data were created or analyzed in this study. Data sharing is not applicable to this article.
